# Look Beyond Plasma Membrane Biophysics: Revealing Considerable Variability of the Dipole Potential Between Plasma and Organelle Membranes of Living Cells

**DOI:** 10.3390/ijms26030889

**Published:** 2025-01-22

**Authors:** Mate Szabo, Bence Cs. Szabo, Kitti Kurtan, Zoltan Varga, Gyorgy Panyi, Peter Nagy, Florina Zakany, Tamas Kovacs

**Affiliations:** Department of Biophysics and Cell Biology, Faculty of Medicine, University of Debrecen, H-4032 Debrecen, Hungary; szabo.mate@med.unideb.hu (M.S.); cs.szabo.bence@med.unideb.hu (B.C.S.); kurtan.kitti@med.unideb.hu (K.K.); veze@med.unideb.hu (Z.V.); panyi@med.unideb.hu (G.P.); nagyp@med.unideb.hu (P.N.)

**Keywords:** membrane biophysics, membrane dipole potential, intracellular organelles, lysosome, Golgi, endoplasmic reticulum, mitochondrion, fluorescence microscopy

## Abstract

Due to the lack of measurement techniques suitable for examining compartments of intact, living cells, membrane biophysics is almost exclusively investigated in the plasma membrane despite the fact that its alterations in intracellular organelles may also contribute to disease pathogenesis. Here, we employ a novel, easy-to-use, confocal microscopy-based approach utilizing F66, an environment-sensitive fluorophore in combination with fluorescent organelle markers and quantitative image analysis to determine the magnitude of the molecular order-related dipole potential in the plasma membrane and intracellular organelles of various tumor and neural cell lines. Our comparative analysis demonstrates considerable intracellular variations of the dipole potential that may be large enough to modulate protein functions, with an inward decreasing gradient on the route of the secretory/endocytic pathway (plasma membrane >> lysosome > Golgi > endoplasmic reticulum), whereas mitochondrial membranes are characterized by a dipole potential slightly larger than that of lysosomes. Our approach is suitable and sensitive enough to quantify membrane biophysical properties selectively in intracellular compartments and their comparative analysis in intact, living cells, and, therefore, to identify the affected organelles and potential therapeutic targets in diseases associated with alterations in membrane lipid composition and thus biophysics such as tumors, metabolic, neurodegenerative, or lysosomal storage disorders.

## 1. Introduction

While the main function of biological membranes was initially thought to be separating compartments from each other to allow for a controlled composition, now it is obvious that lipids in these bilayers also actively modulate the structure and function of membrane proteins, which can generally occur via a continuum of direct lipid–protein interactions and indirect actions mediated through bulk biophysical properties. Recent advances in high-resolution X-ray crystallography and cryo-electron microscopy techniques and molecular dynamics tools with high computation power shifted the focus of researchers to study the direct effects of lipids on proteins, and, consequently, the potential contribution of indirect membrane biophysics-related mechanisms to the modulation of protein functions is largely neglected in spite of the fact that the latter were previously shown to influence various membrane proteins including different channels, transporters, G protein-coupled receptors and receptor tyrosine kinases [1,2,3]. The scarcity of such studies can be at least partially related to the cumbersome nature of biophysical measurement techniques in living cells. Biophysical parameters of membranes are usually examined using techniques that cannot be applied in living cells, such as NMR and ESR spectroscopy [4,5,6], molecular dynamics simulations [7,8], wide-angle X-ray scattering [9], small-angle X-ray/neutron scattering [4,5,6], cryo-electron microscopy [10], or atomic force microscopy [11]. Such properties of living cells can be investigated with environment-sensitive fluorophores such as TMA-DPH (4′-(trimethylammonio)-diphenylhexatriene) [12,13,14,15,16,17], Laurdan (6-dodecanoyl-N,N-dimethyl-2-naphthylamine) [12,13,14,15,18,19,20], PY3174 (4-[2-(6-Dibutylamino-5-fluoro-naphthalen-2-yl)-vinyl]-1-(3-triethylammonio-propyl)-pyridinium dibromide) [15,17,21], or di-8-ANEPPS (4-(2-[6-(dioctylamino)-2-naphthalenyl]ethenyl)-1-(3-sulfopropyl pyridinium inner salt) [12,14,17,22,23,24,25,26,27,28,29,30,31]. While these dyes are invaluable tools for examining membrane biophysics in intact cells, their unfavorable spectral properties limit their applicability in combination with other commonly used fluorophores.

In general, biophysical properties are intrinsically related to the structural arrangement of membrane constituents, which are typically quantified by three order-related parameters, i.e., membrane fluidity, hydration, and dipole potential, in living cells. Although all three are substantially linked to one another, they characterize structural organization at different bilayer depths. Fluidity and hydration depict molecular order at the deeper hydrophobic layers, whereas the dipole potential depicts it at the membrane–water interface [3,32,33,34]. The least known of the three, the dipole potential originates from the non-random organization of molecular dipoles of lipids and water molecules at the interfacial region of bilayers, which results in the generation of a large positive intramembrane potential [3,35,36,37]. Through affecting charged and polar residues, the electric field generated by the dipole potential may modulate conformational transitions and, consequently, the functions of transmembrane proteins, as shown previously in voltage-gated potassium channels [14,38,39], Na^+^-K^+^ ATPase [40], P-glycoprotein [24], ErbB proteins [27], and G-protein coupled receptors [22]. The magnitude of dipole potential is determined by the levels of membrane constituents, mainly that of various phospholipids [31], glucosylceramides and ceramides [14,28], and, in particular, cholesterol [8,12,17,24,25,26,28,29,41]. Accordingly, its value was shown to display lateral heterogeneity in microdomains of the plasma membrane [28,41]. While biophysical properties in general, and dipole potential in particular, have been examined in the plasma membrane by quite a few studies, far less is known about the bilayers of intracellular organelles.

The membranes of intracellular compartments are characterized by a unique lipid composition and, thus, presumably distinctive membrane biophysical properties. For example, within the secretory pathway, membranes of the endoplasmic reticulum and the Golgi apparatus are generally considered to display low packing density with low levels of cholesterol and complex sphingolipids, whereas the plasma membrane is enriched in these lipids to provide mechanical stability to the bilayer. Along the endocytic pathway, early endosomes are expected to display a composition similar to that of the plasma membrane, while lysosomes contain lower levels of cholesterol and several distinctive lipid species. On the other hand, mitochondria comprise unique lipids such as cardiolipin or phosphatidylglycerol typically accompanied by a low abundance of cholesterol [42,43,44]. Although lipidomic studies provided evidence for these distinctive compositions, it is mostly unclear whether and how they are manifested in the altered membrane biophysical properties of living, intact cells. Only sporadic reports demonstrated measurements of biophysical parameters in the endoplasmic reticulum [45,46], Golgi apparatus [47], lysosomes [48], or mitochondria [49,50], and a comprehensive and comparative analysis of these features is still missing in living cells.

The main objective of this study was to optimize a novel, easy-to-use method to investigate differences in the magnitude of dipole potential between the plasma membrane and bilayers of various intracellular organelles of individual, intact, living cells. For this, we utilized F66, a voltage-sensitive 3-hydroxyflavone fluorophore that is characterized by an electric field-modulated, excited-state intramolecular proton transfer (ESIPT) reaction resulting in normal (N*) and tautomer (T*) excited states with well-separated bands in its emission spectrum. As shown by previous studies, the T*/N* emission ratio of the dye shows a strong positive correlation with the magnitude of the dipole potential [17,28,41,51]. We applied F66 in combination with common intracellular organelle markers to selectively examine the dipole potential in the membrane of various intracellular compartments in a quantitative confocal microscopy-based emission ratiometric assay. In various, unrelated cell types, SKBR-3 human breast cancer cells, HeLa human cervical carcinoma cells, and SH-SY5Y human neuroblastoma cells serving as potential models of diseases including various tumors and neurodegenerative disorders in which alterations in lipid levels have pathophysiological relevance, we determined the magnitude of the dipole potential exclusively in the plasma membrane and bilayers of lysosomes, the Golgi apparatus, the endoplasmic reticulum, and mitochondria so that we were able to compare these cellular membranes in terms of membrane biophysics. Using our novel method, we demonstrated an inward decreasing dipole potential gradient in organelles of the secretory/endocytic pathway and also showed that the dipole potential of mitochondrial membranes, albeit being smaller than that of the plasma membrane, is, at first glance, unexpectedly high in spite of its presumably low cholesterol content when compared to the other examined intracellular compartments. In the future, our measurement technique may be utilized to reveal biophysical alterations of intracellular compartments and thus identify important pathophysiological factors and potential therapeutic targets in diseases associated with alterations in membrane lipid composition such as tumors and metabolic, neurodegenerative, or lysosomal storage disorders.

## 2. Results

### 2.1. The Magnitude of Dipole Potential Displays Large Variations in Cellular Membranes

**Figure 1:**
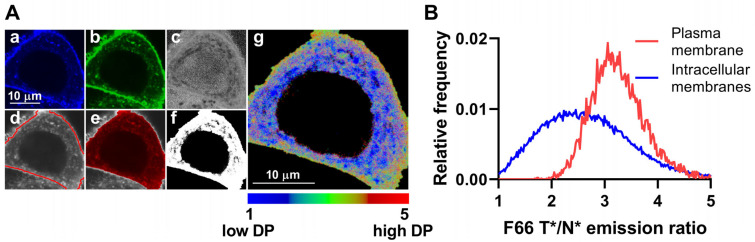
Differences in the magnitude of the F66 emission ratio between the plasma membrane and intracellular membranes. (**A**) SKBR-3 cells grown onto an eight-well chambered coverglass were stained with F66. Representative confocal microscopic images taken at the midplane of cells show F66 intensities of the N* normal (**a**) and T* tautomeric (**b**) excited states and the T*/N* emission ratios calculated on a pixel-by-pixel basis after background subtraction (**c**). During quantitative image analysis, a custom-written manually seeded watershed algorithm was applied to segment images into plasma membrane (**d**) and intracellular pixels (**e**). Pixels corresponding to intracellular membranes were identified based on having an F66 fluorescence intensity above a threshold value (**f**). F66 T*/N* emission ratios of intracellular membrane pixels positively correlating with the magnitude of the dipole potential (‘DP’) were displayed in a representative color-scale image (**g**). (**B**) Representative histograms demonstrate pixelwise distributions of the F66 emission ratio in regions corresponding to the plasma membrane and intracellular membranes.

Given that the magnitude of dipole potential is mainly determined by the lipid composition of bilayers [3,35,37], and various cellular membranes are characterized by large qualitative and quantitative differences in their constituents [42,43,44], it is reasonable to assume that dipole potential may show significant variations throughout the membranes of the cell. In order to support this hypothesis, we applied F66, a voltage-sensitive 3-hydroxyflavone fluorophore, and determined its T*/N* emission ratio in different cellular membranes, which was previously described to positively correlate with the magnitude of membrane dipole potential [17,28,41,51]. We utilized the dye in a double-labeling protocol as we incubated cells in the presence of the fluorophore for 24 h to ensure its accumulation in all intracellular membranes, which was followed by subsequent staining for 10 min for the unequivocal visualization of the plasma membrane. We acquired images from the midplane of SKBR-3 cells in wavelength ranges corresponding to the N* and T* excited states of the dye (Figure 1A, panels a and b, respectively). In accordance with our previous studies [17,28,41], such an application of the dye did not induce any noticeable membrane disruption manifesting in signs of cell damage or even recognizable morphological alterations. During quantitative image analysis, we calculated the F66 T*/N* emission ratio on a pixel-by-pixel basis (Figure 1A, panel c). Next, we applied a custom-written manually seeded watershed algorithm to identify plasma membrane pixels and intracellular areas of individual cells based on the F66 fluorescence intensities (Figure 1A, panels d and e, respectively). Then, we determined an F66 intensity threshold value equal to four times the mean F66 intensity of a cell-free, background area, and intracellular pixels having larger intensity than the threshold were identified as intracellular membrane pixels (Figure 1A, panel f and Supplementary Figure S1). F66 T*/N* emission ratios were subsequently examined in pixels corresponding to the plasma membrane and bilayers of intracellular organelles and displayed on a color scale (Figure 1A, panel g). The representative image demonstrates large variations in the F66 T*/N* emission ratio, that is, the magnitude of the dipole potential, throughout the cell. Namely, the plasma membrane and bilayers found in the peripheral regions of the cell are characterized by a larger dipole potential, as mirrored by the reddish-greenish color of pixels, while, in general, more internal membranes have a smaller dipole potential, which is indicated by the predominantly blue color of these pixels. The difference in the magnitude of the dipole potential between the plasma membrane and intracellular bilayers is also visible in representative F66 T*/N* emission ratio histograms, demonstrating a strong leftward shift in intracellular membrane pixels when compared to plasma membrane pixels (Figure 1B). Furthermore, it is important to note that the dipole potential of intracellular bilayers seems extensively heterogeneous, as illustrated both by the diverse color of intracellular pixels in the representative image (Figure 1A, panel g) and the broadening of the pixelwise distribution of the F66 T*/N* emission ratio (Figure 1B), which may refer to considerable variations in the magnitude of the dipole potential in membranes of various organelles.

### 2.2. The Magnitude of Dipole Potential of Intracellular Organelles Compared with That of the Plasma Membrane

**Figure 2:**
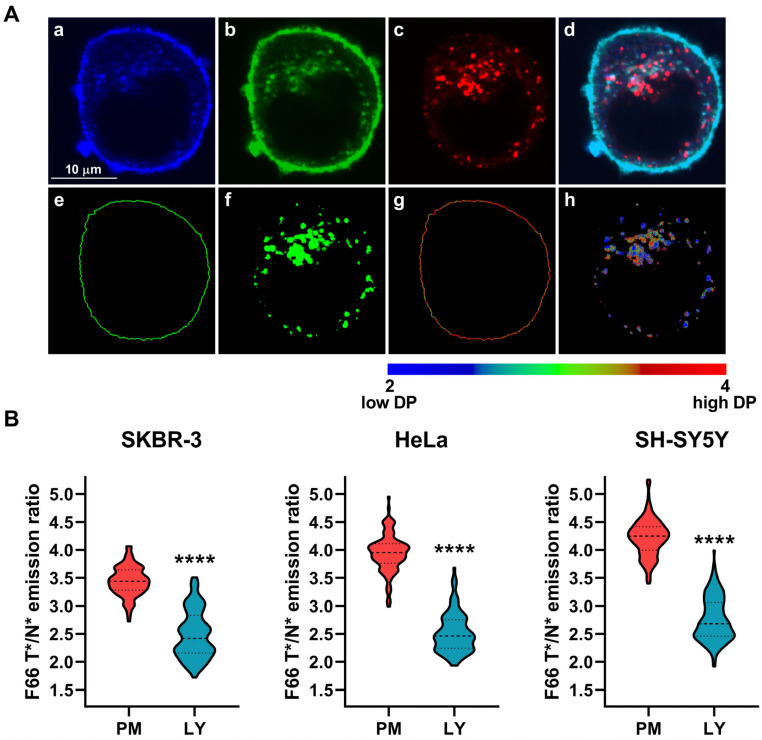
Differences in the magnitude of the F66 emission ratio between the plasma membrane and lysosomal membranes. SKBR-3, HeLa, and SH-SY5Y cells grown onto an eight-well chambered coverglass were labeled with F66 and LysoTracker Deep Red. (**A**) Representative confocal microscopic images taken at the midplane of cells show intensities of the N* normal (**a**) and T* tautomeric (**b**) excited forms of F66 and LysoTracker Deep Red (**c**), while an overlay image is displayed in panel (**d**). During quantitative image analysis, a custom-written manually seeded watershed algorithm was applied to identify plasma membrane pixels (‘PM’, **e**), while pixels corresponding to lysosomal membranes were identified based on their LysoTracker Deep Red fluorescence above a threshold intensity in the intracellular area (‘LY’, **f**). Subsequently, the T*/N* emission ratios positively correlating with the magnitude of the dipole potential (‘DP’) were calculated after background subtraction on a pixel-by-pixel basis and displayed in representative color-scale images separately for plasma membrane (**g**) and lysosomal membrane (**h**) pixels. (**B**) The median F66 T*/N* emission ratio values of individual SKBR-3, HeLa, and SH-SY5Y cells were determined separately from data of pixels corresponding to the plasma membrane and lysosomal membranes. Violin plots were generated from median F66 T*/N* emission ratio values of n = 107–154 individual cells obtained from five independent experiments, which also display median values with quartiles. Asterisks indicate significant differences between plasma and lysosomal membranes (**** *p* < 0.0001, Welch’s *t*-test).

While the analysis described above convincingly suggested that the magnitude of dipole potential of intracellular bilayers is smaller than that of the plasma membrane and shows considerable heterogeneity, it did not give us any information about its values in individual organelles. To examine these, we combined F66 staining and the confocal microscopic dipole potential measurement with the application of fluorescent markers to exclusively identify the different intracellular compartments. In the first step of these experiments, while simultaneously using the F66 labeling protocol described above, we incubated the cells in the presence of LysoTracker™ Deep Red to stain lysosomes and, subsequently, acquired images of the N* and T* excited forms of F66 and the lysosome marker (Figure 2A, panels a–d). During the quantitative image analysis, after identifying plasma membrane pixels and intracellular areas of cells as described above, we defined lysosomal pixels by determining a threshold fluorescence of the organelle marker and considered pixels as lysosomal membranes when having an intensity larger than the threshold value (Figure 2A, panels e and f). Subsequently, we determined the median F66 T*/N* emission ratios separately in pixels corresponding exclusively to the plasma membrane and lysosomal membranes of the same cell (Figure 2A, panels g and h). First, we performed measurements in SKBR-3 human breast cancer cells and observed that lysosomal membranes were characterized by a significantly reduced F66 T*/N* emission ratio when compared to that of the plasma membrane (2.488 ± 0.424 vs. 3.441 ± 0.263, n = 107, *p* < 0.0001) (Figure 2B). While it seems logical that such a difference between plasma membranes and lysosomal bilayers can be a general property of cells of any type, we performed similar measurements in two unrelated cell lines to support this assumption. We found strongly consistent results with reduced lysosomal dipole potential compared to the plasma membrane both in HeLa human cervical carcinoma cells (lysosomes: 2.518 ± 0.351 vs. plasma membrane: 3.947 ± 0.333, n = 115, *p* < 0.0001) and SH-SY5Y human neuroblastoma cells (lysosomes: 2.759 ± 0.391 vs. plasma membrane: 4.224 ± 0.330, n = 154, *p* < 0.0001).

**Figure 3:**
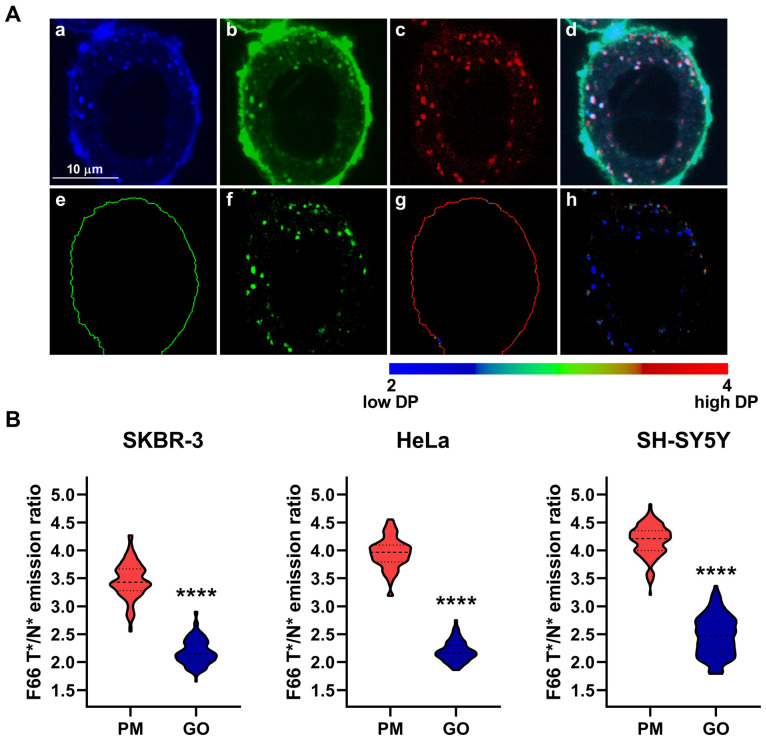
Differences in the magnitude of the F66 emission ratio between the plasma membrane and Golgi membranes. SKBR-3, HeLa, and SH-SY5Y cells grown onto an eight-well chambered coverglass were transduced with the CellLight Golgi-RFP, BacMam 2.0 marker and labeled with F66. (**A**) Representative confocal microscopic images taken at the midplane of cells show intensities of the N* normal (**a**) and T* tautomeric (**b**) excited forms of F66 and Golgi-RFP (**c**), while an overlay image is displayed in panel (**d**). During quantitative image analysis, a custom-written manually seeded watershed algorithm was applied to identify plasma membrane pixels (‘PM’, **e**), while pixels corresponding to Golgi membranes were identified based on their RFP fluorescence above a threshold intensity in the intracellular area (‘GO’, **f**). Subsequently, the T*/N* emission ratios positively correlating with the magnitude of the dipole potential (‘DP’) were calculated after background subtraction on a pixel-by-pixel basis and displayed in representative color-scale images separately for plasma membrane (**g**) and Golgi membrane (**h**) pixels. (**B**) The median F66 T*/N* emission ratio values of individual SKBR-3, HeLa, and SH-SY5Y cells were determined separately from data of pixels corresponding to the plasma membrane and Golgi membranes. Violin plots were generated from median F66 T*/N* emission ratio values of n = 133–138 individual cells obtained from five independent experiments, which also display median values with quartiles. Asterisks indicate significant differences between plasma and Golgi membranes (**** *p* < 0.0001, Welch’s *t*-test).

To further characterize the dipole potential of intracellular membranes, we repeated our measurements after staining the Golgi apparatus by transducing cells using CellLight™ Golgi-RFP, BacMam 2.0. We performed imaging and quantitative image analysis as described above for lysosomes (Figure 3A). In these experiments, pixels corresponding to Golgi membranes displayed significantly reduced F66 T*/N* emission ratios, i.e., a lower dipole potential, when compared to plasma membranes in SKBR-3 (Golgi: 2.178 ± 0.226 vs. plasma membrane: 3.460 ± 0.321, n = 137, *p* < 0.0001), HeLa (Golgi: 2.193 ± 0.182 vs. plasma membrane: 3.952 ± 0.272, n = 133, *p* < 0.0001), and SH-SY5Y cells (Golgi: 2.468 ± 0.359 vs. plasma membrane: 4.170 ± 0.282, n = 138, *p* < 0.0001) (Figure 3B).

**Figure 4:**
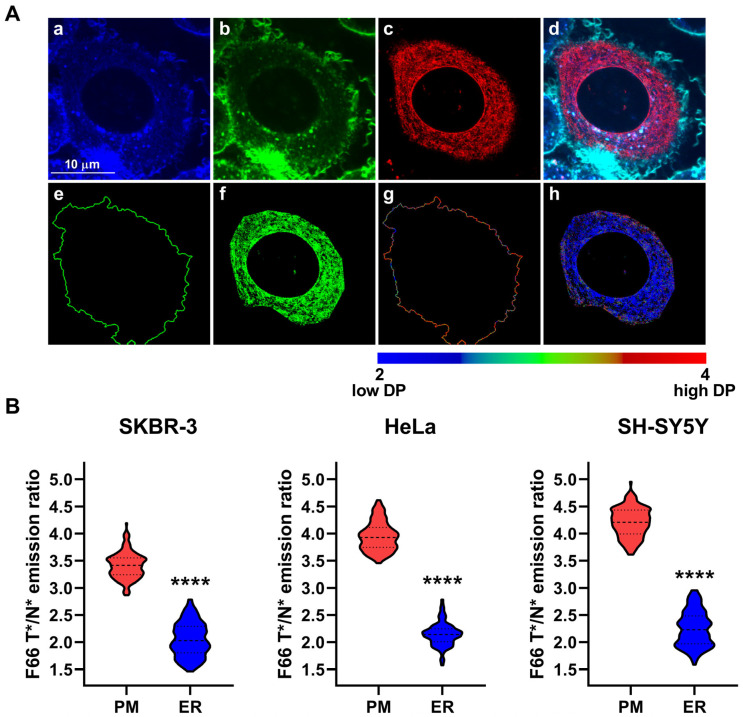
Differences in the magnitude of the F66 emission ratio between the plasma membrane and endoplasmic reticulum membranes. SKBR-3, HeLa, and SH-SY5Y cells grown onto an eight-well chambered coverglass were transduced with the CellLight ER-GFP, BacMam 2.0 marker and labeled with F66. (**A**) Representative confocal microscopic images taken at the midplane of cells show intensities of the N* normal (**a**) and T* tautomeric (**b**) excited forms of F66 and ER-GFP (**c**), while an overlay image is displayed in panel (**d**). During quantitative image analysis, a custom-written manually seeded watershed algorithm was applied to identify plasma membrane pixels (‘PM’, **e**), while pixels corresponding to endoplasmic reticulum membranes were identified based on their GFP fluorescence above a threshold intensity in the intracellular area (‘ER’, **f**). Subsequently, the T*/N* emission ratios positively correlating with the magnitude of the dipole potential (‘DP’) were calculated after background subtraction on a pixel-by-pixel basis and displayed in representative color-scale images separately for plasma membrane (**g**) and endoplasmic reticulum membrane (**h**) pixels. (**B**) The median F66 T*/N* emission ratio values of individual SKBR-3, HeLa, and SH-SY5Y cells were determined separately from data of pixels corresponding to the plasma membrane and endoplasmic reticulum membranes. Violin plots were generated from median F66 T*/N* emission ratio values of n = 114–152 individual cells obtained from five independent experiments, which also display median values with quartiles. Asterisks indicate significant differences between plasma and endoplasmic reticulum membranes (**** *p* < 0.0001, Welch’s *t*-test).

Next, we quantified the dipole potential of membranes of the endoplasmic reticulum after visualizing that organelle by transducing cells with CellLight™ ER-GFP, BacMam 2.0, followed by imaging and analysis as detailed above (Figure 4A). Consistent with our previous experiments, we found significantly lower F66 T*/N* emission ratios, i.e., a reduced dipole potential in bilayers of the endoplasmic reticulum when compared to that observed in the plasma membrane in SKBR-3 (endoplasmic reticulum: 2.047 ± 0.310 vs. plasma membrane: 3.414 ± 0.240, n = 152, *p* < 0.0001), HeLa (endoplasmic reticulum: 2.143 ± 0.213 vs. plasma membrane: 3.946 ± 0.265, n = 114, *p* < 0.0001), and SH-SY5Y cells (endoplasmic reticulum: 2.246 ± 0.321 vs. plasma membrane: 4.207 ± 0.270, n = 139, *p* < 0.0001) as well (Figure 4B).

**Figure 5:**
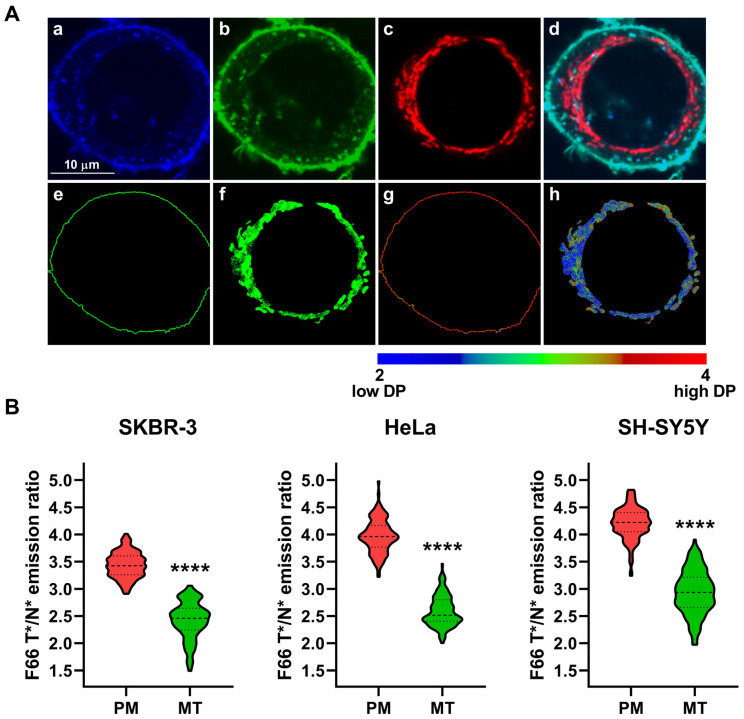
Differences in the magnitude of the F66 emission ratio between the plasma membrane and mitochondrial membranes. SKBR-3, HeLa, and SH-SY5Y cells grown onto an eight-well chambered coverglass were labeled with F66 and MitoTracker Deep Red. (**A**) Representative confocal microscopic images taken at the midplane of cells show intensities of the N* normal (**a**) and T* tautomeric (**b**) excited forms of F66 and MitoTracker Deep Red (**c**), while an overlay image is displayed in panel (**d**). During quantitative image analysis, a custom-written manually seeded watershed algorithm was applied to identify plasma membrane pixels (‘PM’, **e**), while pixels corresponding to mitochondrial membranes were identified based on their MitoTracker Deep Red fluorescence above a threshold intensity in the intracellular area (‘MT’, **f**). Subsequently, the T*/N* emission ratios positively correlating with the magnitude of the dipole potential (‘DP’) were calculated after background subtraction on a pixel-by-pixel basis and displayed in representative color-scale images separately for plasma membrane (**g**) and mitochondrial membrane (**h**) pixels. (**B**) The median F66 T*/N* emission ratio values of individual SKBR-3, HeLa, and SH-SY5Y cells were determined separately from data of pixels corresponding to the plasma membrane and mitochondrial membranes. Violin plots were generated from median F66 T*/N* emission ratio values of n = 108–135 individual cells obtained from five independent experiments, which also display median values with quartiles. Asterisks indicate significant differences between plasma and mitochondrial membranes (**** *p* < 0.0001, Welch’s *t*-test).

In the last part of our measurements, we investigated the dipole potential in mitochondria after visualizing them by incubating the cells in the presence of MitoTracker™ Deep Red and carrying out image acquisition and analysis as above (Figure 5A). In accordance with data obtained in lysosomes, the Golgi apparatus, and the endoplasmic reticulum, the membranes of mitochondria were also characterized by a lower dipole potential mirrored by F66 T*/N* emission ratios smaller than that obtained in the plasma membranes in all examined cell types including SKBR-3 (mitochondria: 2.421 ± 0.342 vs. plasma membrane: 3.438 ± 0.236, n = 110, *p* < 0.0001), HeLa (mitochondria: 2.588 ± 0.282 vs. plasma membrane: 3.975 ± 0.309, n = 108, *p* < 0.0001), and SH-SY5Y (mitochondria: 2.938 ± 0.405 vs. plasma membrane: 4.206 ± 0.290, n = 135, *p* < 0.0001) (Figure 5B).

### 2.3. Comparison of the Magnitude of Dipole Potential in Different Intracellular Organelles

**Figure 6:**
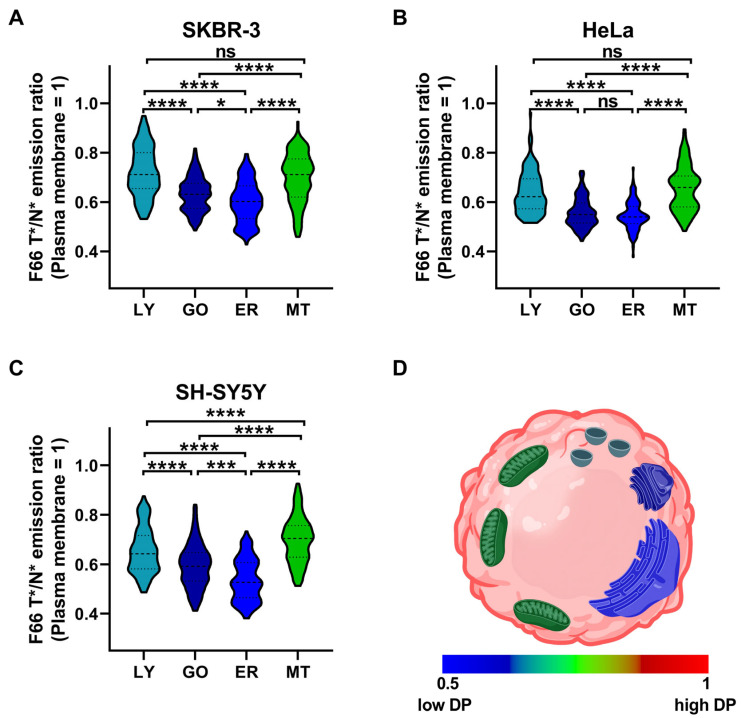
Differences in the magnitude of the F66 emission ratio between the various cellular membranes. SKBR-3, HeLa and SH-SY5Y cells grown onto an eight-well chambered coverglass were labeled with F66 and LysoTracker Deep Red, CellLight Golgi-RFP BacMam 2.0, CellLight ER-GFP BacMam 2.0, or MitoTracker Deep Red, which was followed by quantification of the median F66 T*/N* emission ratio positively correlating with the magnitude of the dipole potential (‘DP’) separately for pixels corresponding to the plasma membrane and the membranes of the labeled lysosomes (‘LY’), Golgi (‘GO’), endoplasmic reticulum (‘ER’), and mitochondria (‘MT’) in each individual cell. Subsequently, the median F66 T*/N* ratio of the organelle membrane was normalized to the value determined in the plasma membrane of the given cell. Violin plots were generated from the normalized median F66 T*/N* emission ratio values of n = 107–154 individual SKBR-3 (**A**), HeLa (**B**), and SH-SY5Y (**C**) cells obtained from five independent experiments, which also display median values with quartiles. Asterisks indicate significant differences between membranes of the different intracellular organelles (* *p* < 0.05, *** *p* < 0.001, and **** *p* < 0.0001, ANOVA followed by Tukey’s HSD test). (**D**) The colors of bilayers in a schematic cell illustrate differences in the relative magnitude of the dipole potential in the plasma membrane and the membranes of lysosomes, Golgi, endoplasmic reticulum, and mitochondria, which were determined as the median normalized F66 T*/N* emission ratios obtained from the three examined cell types. The panel was created in Biorender (https://BioRender.com/g58o031 (accessed on 14 January 2025)).

When comparing the magnitude of the membrane dipole potential of intracellular organelles, we employed an alternative evaluation method on data obtained in the experiments described above. Namely, we normalized the median F66 T*/N* emission ratios of organelle membranes to the median value of the ratio in the plasma membrane of the same cell to determine relative differences between intracellular regions. When analyzing our data with this method, we found that the magnitude of the dipole potential showed an inwards decreasing gradient along the secretory/endocytic pathway from the plasma membrane to the endoplasmic reticulum (Figure 6). The highest normalized F66 T*/N* emission ratios, that is, magnitudes of the dipole potential, were found in the plasma membrane, which decreased in the order of lysosome > Golgi apparatus > endoplasmic reticulum in all examined cell types including SKBR-3 (lysosomes: 0.722 ± 0.101, n = 107; Golgi: 0.633 ± 0.070, n = 137; and endoplasmic reticulum: 0.600 ± 0.084, n = 152), HeLa (lysosomes: 0.640 ± 0.086, n = 115; Golgi: 0.558 ± 0.061, n = 133; and endoplasmic reticulum: 0.545 ± 0.058, n = 114), and SH-SY5Y (lysosomes: 0.655 ± 0.093, n = 154; Golgi: 0.594 ± 0.089, n = 138; and endoplasmic reticulum: 0.536 ± 0.084, n = 128). All differences between these organelles were found statistically significant except for that between the Golgi apparatus and the endoplasmic reticulum in HeLa cells. The normalized F66 T*/N* emission ratios observed in mitochondrial membranes were in general similar to that observed in lysosomes in SKBR-3 (0.698 ± 0.100, n = 110) and HeLa cells (0.655 ± 0.091, n = 108), whereas it was significantly larger than that of lysosomes in SH-SY5Y cells (0.699 ± 0.093, n = 135). In summary, all examined cell lines exhibited the following similar pattern in the magnitude of the membrane dipole potential: plasma membrane >> mitochondrion ≥ lysosome > Golgi apparatus > endoplasmic reticulum.

## 3. Discussion

In this study, we optimized a confocal microscopy- and quantitative image analysis-based emission ratiometric method utilizing F66, a voltage-sensitive fluorophore with favorable spectral properties, in combination with fluorescent intracellular organelle markers to quantitate dipole potential, a major order-related membrane biophysical parameter, separately in various cellular membranes of intact, living cells. Our novel technique allowed us to reach the following major conclusions: (i) the magnitude of the dipole potential is the largest in the plasma membrane in all examined cell types including SKBR-3 human breast cancer cells, HeLa human cervical carcinoma cells, and SH-SY5Y human neuroblastoma cells (Figure 2, Figure 3, Figure 4, Figure 5 and Figure 6). (ii) The dipole potential shows notable heterogeneity in intracellular membranes (Figure 1). (iii) When examining organelles of the secretory/endocytic pathway, the magnitude of the dipole potential shows an inwards decreasing gradient in the order of plasma membrane >> lysosome > Golgi apparatus > endoplasmic reticulum, and a strongly similar pattern of relative magnitudes is displayed in all cell types (Figure 2, Figure 3, Figure 4 and Figure 6). (iv) The dipole potential of mitochondria is more variable among the examined cell lines, but it is typically closest to that of lysosomes (Figure 5 and Figure 6). (v) The similar pattern of the dipole potential distribution among various cellular bilayers of three unrelated cell types suggests that these differences are expected to be common characteristics of human cells. (vi) Our method may be a useful tool to identify organelles in which notable membrane biophysical changes occur in diseases with altered membrane lipid levels.

In spite of their presumable biological relevance, the indirect, biophysics-related effects of lipids have been commonly neglected in recent studies, at least partially due to limitations in the examination of membrane biophysical properties in living cells. A limited number of environment-sensitive fluorophores can be applied for this purpose in assays quantifying changes in their fluorescence-associated parameters as a function of their local microenvironment, i.e., typically the molecular order and polarity in the vicinity of their fluorescent moiety [33,52,53]. From among the most widely utilized fluorophores, the fluorescence anisotropy of TMA-DPH reports on membrane fluidity, i.e., the motional freedom in the deep hydrophobic layers of bilayers [12,13,14,15,16,17], while the generalized polarization of Laurdan mirrors membrane hydration, that is, the extent of penetration of water molecules into the hydrophobic regions [12,13,14,15,18,19,20]. The main disadvantage of these fluorophores, and many other similar probes, is their low photostability and excitation falling in the UV range. As a result, they are not suitable for conventional microscopic imaging but rather examined mainly with spectrofluorometry or, in certain cases, two-photon microscopy [13,19,54,55]. Conventional imaging can be performed by quantifying the generalized polarization of PY3174 to estimate membrane hydration [15,17,21] or the excitation ratio of di-8-ANEPPS to determine dipole potential [12,14,17,22,23,24,25,26,27,28,29,30,31]. While the latter fluorophore and many related probes, such as the commonly applied di-4-ANEPPDHQ [56,57], are suitable for imaging with conventional lasers and optical setups, their wide excitation and emission spectra make their combination with other widely used fluorophores difficult. Di-8-ANEPPS can be considered the most widely applied fluorophore suitable for following changes in the magnitude of dipole potential in living cells. Its application is based on electrochromism, that is, changes in its spectral properties depending on the strength of the local electric field due to an interaction of the electric field with the ground-state and excited-state dipole moments of the chromophore group. The dye can be utilized most reliably in an excitation ratiometric assay when the dye is excited at two different wavelengths and its emission is measured in the same range at the red edge of its fluorescence spectrum. While di-8-ANEPPS was successfully used in a variety of studies, its applicability is strongly limited in combination with other widely utilized fluorophores such as GFP or RFP because of their large spectral overlap [12,14,17,22,23,24,25,26,27,28,29,30,31]. F66, a dipole potential-sensitive 3-hydroxyflavone dye, represents an interesting compound that can act as a di-8-ANEPPS alternative and efficiently report on membrane biophysics due to its ESIPT reaction resulting in normal (N*) and tautomer (T*) excited states with well-separated bands in its emission spectrum. In F66 and several related designed analogs, while the N* and T* emission bands display modest electrochromic and solvatochromic shifts, their relative intensities may exhibit large variations since the ESIPT reaction, and consequently, the relative abundance of the two species is substantially modulated by the strength of the local electric field. This occurs due to a large difference in the electronic charge separation, that is, the dipole moment, between the N* and T* forms. Namely, while the N* is characterized by a large dipole moment, that of the T* form is very small. As a result, changes in the magnitude of the local electric field may substantially alter the energy of the N* state while only marginally influencing that of the T*, which results in a relative stabilization or destabilization of the N* state when compared to T*. In accordance, the local electric field influences the distribution of excited species between the N* and T* states, leading to subsequent changes in their relative emission [58,59]. Consistently, the T*/N* emission ratio of F66 was previously demonstrated to display a strong positive correlation with the magnitude of the dipole potential [17,28,41,51]. The main advantage of F66 is its excitability in the near-UV range (405 nm, a commonly used laser line) and, as a result, its excitation spectrum being well-separated from that of other widely utilized fluorophores such as GFP, RFP, or red-far red dyes, for example, conjugated to intracellular organelle markers.

Within the cell, each compartment is surrounded by a membrane of unique lipid composition due to the topographically restricted synthesis of lipids, their distinctive local metabolism, and selective inter-organelle transport using vesicles or direct contact sites. Most lipids such as phospholipids, cholesterol, and ceramides as precursors for complex sphingolipids, are synthesized in the endoplasmic reticulum, however, cholesterol and sphingolipids are rapidly transported to other organelles. Consequently, bilayers of the endoplasmic reticulum are expected to display loose packing that may be favorable for the insertion and transport of newly synthesized lipids and proteins. The Golgi apparatus, acting as a lipid-based sorting station in the secretory pathway and characterized by an ‘intermediate lipidome’, also represents a site for significant lipid synthesis, however, typically for more complex sphingolipids such as sphingomyelin, glucosylceramides, and higher-order glycosphingolipids that are primarily destined for the plasma membrane. The plasma membrane, serving as a barrier towards the extracellular space, is rather built for stability containing large amounts of sphingolipids and sterols, resulting in a much higher packing density and mechanical stress resistance. In the endocytic pathway, early endosomes are expected to display a composition similar to that of the plasma membrane, whereas inner elements of the pathway such as lysosomes display more different levels of constituents, e.g., gradually reduced amounts of cholesterol and sphingolipids. Mitochondrial membranes display a unique composition in certain aspects reminiscent of those of bacteria, including high levels of the unique cardiolipin, the abundant presence of phosphatidylglycerol, a high phosphatidylethanolamine/phosphatidylcholine ratio, and generally low levels of cholesterol. Although a distinctive set of lipid-synthesizing enzymes is present in mitochondria, there is a considerable lipid exchange at endoplasmic reticulum–mitochondria contact sites [42,43,44,60]. While these unique bilayer compositions are expected to be mirrored in terms of membrane biophysics, such properties of intracellular organelles are scarcely documented, particularly in intact, living cells, as most experiments were previously performed in isolated organelles or model membranes mimicking the composition of a certain compartment. In general, the examination of organelles in living cells can be performed according to four major strategies. (i) First, the lateral motional freedom of a fluorophore showing preferential accumulation in a given organelle can give information about properties of that given bilayer, as was shown by changes in lateral diffusion of GFP-conjugated endoplasmic reticulum resident lipids or proteins [45,46] and TMRM in mitochondria [50]. (ii) Second, an environment-sensitive fluorophore may also localize into a given organelle if conjugated to an appropriate molecular moiety. Such an approach was previously applied to demonstrate increased structural plasticity of the Golgi membrane relative to the more rigid plasma membranes following the spectral relaxation of a Golgi-specific C_6_-NBD-ceramide probe [47], to reveal dynamic coupling between mitochondrial inner membrane fluidity and cellular respiration using fluorescence lifetime imaging on a molecular-rotor [49] and to show fluidity changes in lysosomal membranes during lysosomal fission and fusion using an amine-linked, dual-emissive polarity-responsive probe [48]. The common drawback of these methods is that they can be utilized only for one specific organelle, lacking the possibility of a comparative analysis. (iii) Third, an environment-sensitive fluorophore can be systematically modified to direct it to various organelles so that the properties of those compartments can be directly compared to each other. Such a highly promising approach was applied to generate organelle-targeted c-Laurdan molecules [61] or compounds based on the solvatochromic dye Nile Red, bearing ligands for the specific targeting of the endoplasmic reticulum, mitochondria, lysosomes, Golgi apparatus, plasma membranes, and lipid droplets [53,62,63]. (iv) A simple alternative to the third approach, which was used in the current study, is to combine an environment-sensitive fluorophore accumulating in all cellular membranes with favorable spectral characteristics, in our case F66, with fluorescent organelle markers. Then, quantitative image analysis may differentiate between the compartments to exclusively determine the biophysical parameter in that region. The main advantage of this method, as opposed to those described in point (iii), is that organelles of the same cells can be compared.

Here, using a conventional confocal microscope setup and quantitative image analysis, we applied F66 simultaneously with widely accepted organelle markers carrying commonly used fluorophores, including GFP, RFP, and Deep Red. We were able to demonstrate that while all intracellular membranes exhibit a lower F66 T*/N* emission ratio corresponding to a lower dipole potential, i.e., a smaller extent of molecular order at the membrane–water interface, bilayers of the different compartments show considerable variety. In accordance with the previously reported cholesterol levels in the organelles [42,43,44,60] and the well-known effects of cholesterol to elevate dipole potential due to its intrinsic dipole moment, condensation of the phospholipid headgroup region and changing the amount and orientation of membrane-associated water molecules [8,12,17,24,25,26,28,29,41], we found an inward decreasing dipole potential gradient following the same order as cholesterol abundance: plasma membrane >> lysosome > Golgi apparatus > endoplasmic reticulum (Figure 6). Notably, in all examined cell types, the dipole potential of mitochondrial membranes was found to be significantly lower than that of the plasma membrane but, somewhat unexpectedly based on their presumably low cholesterol abundance, significantly higher than that of the Golgi apparatus and the endoplasmic reticulum, and, in SH-SY5Y cells, even the lysosomes. Given that the application of F66 is based on the intramolecular proton transfer in the excited state, one could argue that this reaction might be sensitive to the environmental pH in the surrounding aqueous phases, and in that case, considering the distinct pH of intracellular organelles such as the lysosomes, the observed differences could be explained simply by these altered pH values. However, this seems unlikely since the molecular moieties involved in the reaction are localized inside the membrane where free protons are not expected to occur. Accordingly, in agreement with previous results obtained with F66 analogs in model vesicles demonstrating no changes in spectroscopic properties in the pH range 5 to 9 [59], we detected no significant difference in the F66 T*/N* emission ratio in cells between pH values of 5.5 and 7.5 (Supplementary Figure S2). While Förster Resonance Energy Transfer (FRET) theoretically enabled by the spectral overlap between F66 and GFP or RFP might undermine our results, such a confounding effect is highly unlikely due to the much higher abundance of the ‘donor’ F66 when compared to the ‘acceptors’ GFP or RFP, which is obvious from intensities of the fluorophores observed in microscopic images and the low probability of colocalization in FRET distance between the membrane-embedded F66 and the mobile GFP or RFP localized in the aqueous phase. Our results, demonstrating notable differences between the dipole potential of various intracellular organelles, are in excellent agreement with the lipid order parameters determined in cellular bilayers of HeLa-derived KB cells with the application of organelle-targeted Nile Red dyes [62] and generalized polarization values obtained in compartments of HeLa cells with organelle-targeted c-Laudan compounds [61], which can be considered as an independent validation of our method. Our results (Figure 6) and those found by others [61,62], demonstrating a relatively high molecular order and dipole potential in the mitochondrial membranes compared to that of other intracellular organelles, might be surprising at first glance due to the previously suggested low cholesterol abundance. However, these findings can be explained by elevated mitochondrial cholesterol levels in certain steroid hormone-producing cells and neurons and the increased order of the inner mitochondrial membrane due to its high protein and cardiolipin contents [64,65].

Conformational changes of transmembrane proteins leading to their different functional states occur with the permission and cooperativity of the surrounding bilayer and, consequently, they are prone to modulatory actions of membrane lipids. Besides direct binding, lipids affect proteins by modifying the bulk biophysical properties of their surroundings [1,2,3]. Molecular order in the deeper layers, i.e., fluidity and hydration, can influence the state of aggregation, rate of conformational changes, secondary structure, and relative stability of certain protein conformers [3,32,66,67], while the large electric field generated by the ordered array of intramolecular dipoles at the membrane–water interface, the dipole potential, may also influence conformational stabilities and thus transition via interaction with polar or charged residues [3,35,36,37]. For example, the magnitude of dipole potential was suggested to influence the voltage-sensing and activation gating of voltage-gated potassium channels [14,38,39], the ATPase and pumping activity of Na^+^-K^+^ ATPase [40] and P-glycoprotein [24], ligand binding, association, autophosphorylation, and the downstream signaling of ErbB proteins [27] and serotonin receptors [22]. In cellular studies, the magnitude of the changes in the F66 emission ratio that were associated with dipole potential alterations capable of inducing biological effects was between 10% and 40% [17,28,41]. In our current experiments, the relative differences between the intracellular organelles and the plasma membrane were ≈25–45%, which supports that the variation in the dipole potential throughout cellular membranes may carry biological relevance, as the proteins are expected to display functional alterations depending on their exact localization. However, this hypothesis has to be tested in the future.

In summary, we have optimized a novel approach that is easy to implement on most conventional confocal microscopes to investigate membrane biophysical properties of intracellular organelles in intact, living, individual cells. The biological relevance of this measurement technique stems from its potential applicability to reveal biophysical alterations in a certain organelle in a disease. Given that membrane biophysical properties in general, and dipole potential in particular, are substantially determined by the amounts of membrane lipids such as various phospholipids [31], glucosylceramides and ceramides [14,28], and cholesterol [8,12,17,24,25,26,28,29,41], their altered levels may lead to changes in biophysical features of bilayers. Such lipid alterations are widely described in human pathologies, including metabolic diseases [68,69,70], neurodegenerative [71,72], lysosomal storage disorders [73], and tumors [33,74,75,76]. Furthermore, abnormalities of intracellular organelles may essentially contribute to the pathomechanism of these conditions. Accordingly, the elucidation of biophysical alterations in intracellular organelles would result in a better understanding of pathophysiology, the identification of therapeutic targets, and the development of novel therapeutic approaches in these diseases in the future, and the development of easy-to-use environment-sensitive fluorophores and measurement techniques, such as the one described here, can be invaluable tools in the future for this purpose.

## 4. Materials and Methods

### 4.1. Cell Lines

The human breast cancer cell line SKBR-3, the human cervical carcinoma cell line HeLa, and the human neuroblastoma cell line SH-SY5Y were obtained from the American Type Culture Collection (Manassas, VA, USA) and grown according to their specifications. For the confocal microscopy measurements, cells were grown on eight-well chambered coverglass (ibidi, Gräfelfing, Germany).

### 4.2. Labeling Cells with Intracellular Organelle Markers and the Dipole Potential-Sensitive F66 Fluorophore

For the visualization of the endoplasmic reticulum and the Golgi apparatus, SKBR-3, HeLa, and SH-SY5Y cells were transduced with CellLight™ ER-GFP, BacMam 2.0, and CellLight™ Golgi-RFP, BacMam 2.0 (both from Thermo Fisher Scientific, Waltham, MA, USA) at 30 particles per cell dissolved into the cell culture media, which was followed by a 24 h incubation period at 37 °C to express the constructs. Alternatively, for labeling lysosomes and mitochondria, cells were incubated with 50 nM LysoTracker™ Deep Red or MitoTracker™ Deep Red FM (both from Thermo Fisher Scientific) for 45 min at 37 °C. For staining cells with the dipole potential-sensitive F66 (N-[3-(40-dihexylamino-3-hydroxy-flavonyl-6-oxy)-propyl]N,N-dimethyl-N-(3-sulfopropyl)-ammonium inner salt, a kind gift from Andrey Klymchenko (Université de Strasbourg, Strasbourg, France)), 10 nM of the dye was applied using a double labeling protocol. First, cells were incubated in the presence of F66 for 24 h to ensure the accumulation of the fluorophore into all intracellular membranes, and second, F66 was additionally given in the last 10 min of incubation for the unequivocal visualization of the plasma membrane. The 10 min F66 staining and imaging were performed in normal Ringer’s solution (in mM: 145 NaCl, 5 KCl, 10 HEPES, 5.5 glucose, 2.5 CaCl_2_ and 1 MgCl_2_, pH 7.35). For the examination of the pH dependence of F66, the 10 min F66 staining and imaging of cells were performed in cellular calibration pH buffers with pH values ranging between 5.5 and 7.5 in the presence of 10 μM valinomycin and 10 μM nigericin to ensure equilibration of extra- and intracellular pH as in our previous study [77].

### 4.3. Confocal Microscopic Imaging of Cells

After labeling and washing, images were acquired at the midplane of cells using an LSM880 confocal laser-scanning microscope (Carl Zeiss AG, Jena, Germany). F66 fluorescence was measured with a ratiometric assay previously shown to be responsive to changes in the membrane dipole potential [17,28,41]. The fluorophore was excited at 405 nm, and its emission was detected in two different wavelength ranges, 463–527 and 543–589 nm, corresponding to the normal (N*) and tautomeric (T*) forms of its excited state, respectively. Sequentially, the fluorescence signals of the organelle markers were recorded from the same cells. Green fluorescent protein (GFP), red fluorescent protein (RFP), and the Deep Red fluorophore were excited at 488, 543, and 633 nm, respectively, and their emitted intensities were measured in the wavelength ranges of 495–550, 570–640, and 650–735 nm, respectively. A single image consisted of 512 × 512 pixels with a pixel size of 52 nm, while the pixel dwell time was 8.24 µs, resulting in a total scanning time of 2.16 s in a single track.

### 4.4. Quantitative Image Analysis

During image analysis performed in Matlab 2024a (Mathworks, Natick, MA, USA), a custom-written manually seeded watershed algorithm was applied to identify plasma membrane pixels and intracellular areas of individual cells based on the F66 fluorescence intensities. In the same individual cells, an intensity threshold value of the fluorescence organelle marker was determined using the maxentropy algorithm confirmed by visual inspection, and pixels of the intracellular area having larger marker intensity than the threshold were identified as membrane pixels corresponding to the given organelle. The process of image segmentation and application of the maxentropy algorithm is demonstrated on a representative cell in Supplementary Figure S3. Subsequently, the F66 T*/N* emission ratio positively correlated with the magnitude of the dipole potential [17,28,41] was calculated after background subtraction on a pixel-by-pixel basis, which was followed by determination of the median F66 T*/N* emission ratios separately in areas corresponding exclusively to the plasma membrane and the membranes of the given organelle of each cell. Furthermore, median F66 T*/N* emission ratios of organelle membranes normalized to the median value of the ratio in the plasma membrane of the same cell were also calculated for an analysis comparing relative differences between intracellular regions.

### 4.5. Statistical Analysis

Measured data are generally represented on violin plots displaying median values with quartiles, which were generated from median F66 T*/N* emission ratio values of *n* = 107–154 individual cells of normal morphology obtained from five independent experiments. The *p* values were calculated by Welch’s *t*-test in analyses displayed in Figure 2, Figure 3, Figure 4 and Figure 5 to compare F66 ratios of the plasma membrane and the given organelle, or by Tukey’s HSD test, which was carried out after significant differences were obtained for between-group effects in ANOVA in the analysis shown in Figure 6 to compare F66 ratios of the different intracellular organelles. Differences were considered significant when *p* < 0.05 (* *p* < 0.05, ** *p* < 0.01, *** *p* < 0.001, **** *p* < 0.0001). Statistical analyses were carried out in GraphPad Prism 8.0 (La Jolla, CA, USA).

## Data Availability

The original contributions presented in the study are included in the article, further inquiries can be directed to the corresponding authors.

## Supplementary Materials

The following supporting information can be downloaded at: https://www.mdpi.com/article/10.3390/ijms26030889/s1.
