# Supplementary material for: Look Beyond Plasma Membrane Biophysics: Revealing Considerable Variability of the Dipole Potential Between Plasma and Organelle Membranes of Living Cells
Source: Int J Mol Sci. 2025 Jan 22;26(3):889. doi: 10.3390/ijms26030889 (PMC11816637; doi:10.3390/ijms26030889)
Supplement: Supplementary file 1 [file ijms-26-00889-s001.zip › ijms-3399432-supplementary.pdf]

Supplementary Material

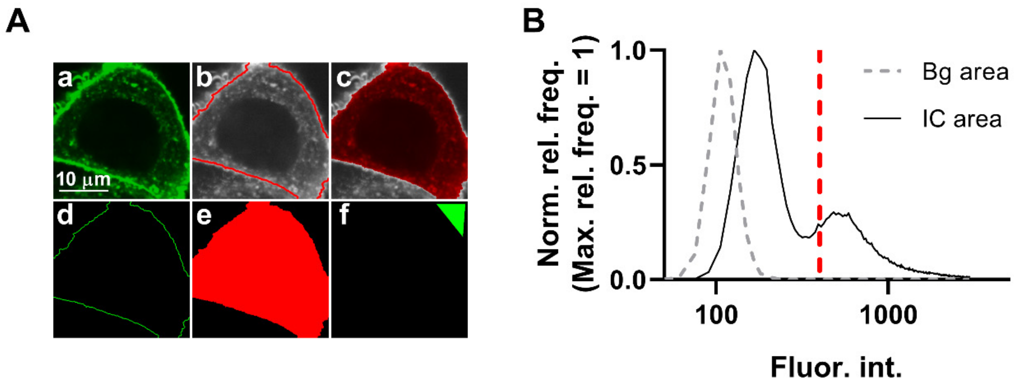

Supplementary Figure S1. Identification of intracellular membranes based on the F66 intensity. (A) SKBR-3 cells grown onto an 8-well chambered coverglass were stained with F66. Representative confocal microscopic images taken at the midplane of cells show F66 intensity of the T* tautomeric excited state (a). During quantitative image analysis, a custom-written manually seeded watershed algorithm was applied to segment images into plasma membrane (b) and intracellular pixels (c) defining a plasma membrane mask (d) and an intracellular area mask (e, 'IC area'). Furthermore, a region was manually drawn in the extracellular space to define an area to determine the background intensity (f, 'Bg area'). (B) Representative histograms demonstrate pixelwise distributions of the fluorescence intensity corresponding to the F66 T* excited forms in the intracellular area ('IC area') and in the extracellular space ('Bg area'). Based on the curves, a threshold fluorescence intensity was determined (at a value 4 times the average intensity of the 'Bg area') and pixels were identified as intracellular membrane pixels for further analysis having an intensity above the threshold value.

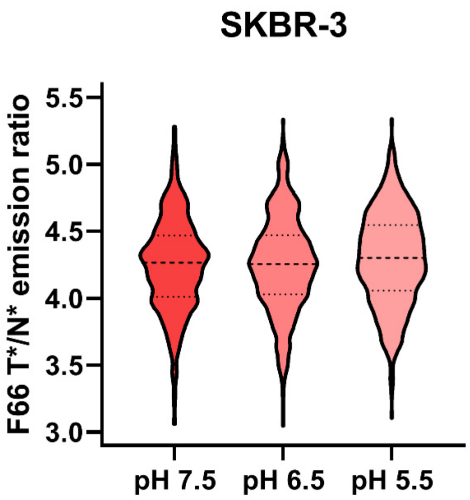

Supplementary Figure S2. Examination of pH dependence of the F66 emission ratio in the plasma membrane. SKBR-3 cells grown onto an 8-well chambered coverglass were labeled with F66 dissolved into cellular calibration pH buffers having pH values ranging from 7.5 to 5.5 in the presence of valinomycin and nigericin to ensure equilibration of extra- and intracellular pH. Images were taken at the midplane of cells to determine fluorescence intensities of the N* normal and T* tautomeric excited forms of F66. During quantitative image analysis, a custom-written manually seeded watershed algorithm was applied to identify plasma membrane pixels. Subsequently, the T*/N* emission ratios positively correlating with the magnitude of the dipole potential were calculated after background subtraction on a pixel-by-pixel basis. The median F66 T*/N* emission ratio values of individual cells were determined from data of pixels corresponding to the plasma membrane. Violin plots were generated from median F66 T*/N* emission ratio values of n = 497–623 individual cells obtained from three independent experiments, which also display median values with quartiles. No significant differences were observed with ANOVA implying that the F66 T*/N* emission ratio does not significantly depend on the surrounding pH.

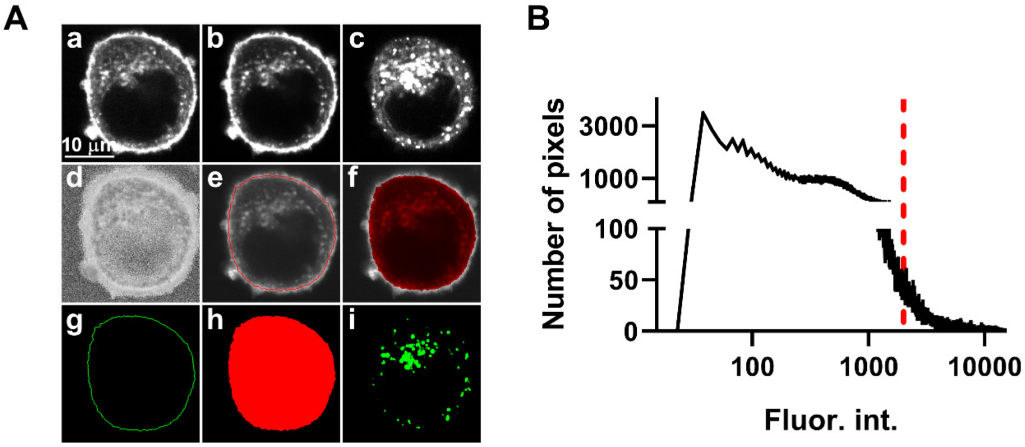

Supplementary Figure S3. A representative example for identification of organelle membranes based on the fluorescence intensity of organelle-specific markers. SKBR-3 cells grown onto an 8-well chambered coverslip were stained with F66 and LysoTracker Deep Red. (A) Representative confocal microscopic images taken at the midplane of cells show intensities of the N* normal (a) and T* tautomeric (b) excited forms of F66, and LysoTracker Deep Red (c). During quantitative image analysis, the T*/N* emission ratios positively correlating with the magnitude of the dipole potential were calculated after background subtraction on a pixel-by-pixel basis (d). Furthermore, a custom-written manually seeded watershed algorithm was applied to segment images into plasma membrane (e) and intracellular pixels (f) defining a plasma membrane mask (g) and an intracellular area mask (h). Based on the LysoTracker Deep Red image (c) a maxentropy algorithm applied in the intracellular region was used to determine a fluorescence intensity threshold and pixels were identified as pixels corresponding to membranes of the given organelle for further analysis having an intensity above the threshold value (i). (B) A representative histogram demonstrates pixelwise distribution of the fluorescence intensity of the organelle marker in the intracellular area and the red line demonstrates the threshold intensity given by the maxentropy algorithm above which pixels were identified as pixels corresponding to the membranes of the given organelle. Subsequently, the median F66 T*/N* emission ratio values of individual cells were determined separately from data of pixels corresponding to the plasma membrane and organelle membranes.
